# Cultural participation rights as a proposed pathway for internal migrant mental health: an institutional analysis from China

**DOI:** 10.3389/fpubh.2026.1866633

**Published:** 2026-07-20

**Authors:** Jie Zhang, Jun Li

**Affiliations:** Law School, Xinjiang University, Urumqi, Xinjiang, China

**Keywords:** cultural participation, internal migrants, legal normative response, mental health, public culture

## Abstract

The mental health risks of internal migrants have become increasingly prominent, yet traditional diagnosis and treatment pathways face structural barriers including the uneven distribution of medical resources, insufficient service accessibility, high time costs, and the stigma surrounding mental health that prevent them from adequately addressing the mental health problems of this population. Cultural participation, as a low-threshold, low-stigma daily practice, may provide preventive support before mental health conditions deteriorate through fostering social connection and cultivating a sense of belonging and identity. Although public cultural services are not designed exclusively for internal migrants, their universally accessible and daily-embedded institutional characteristics may offer this population an alternative pathway for mental health support that complements the barriers to formal help-seeking. The Public Cultural Service Guarantee Law of the People’s Republic of China, as the foundational legislation in this field, establishes a normative framework centered on guaranteeing basic cultural rights and interests, enriching spiritual and cultural life, and advocating healthy and civilized activities. Thus it provides normative space for public cultural services to undertake health promotion functions. In practice, however, resource allocation and performance assessment remain heavily oriented toward physical indicators such as the number of venues, facility size, and event frequency, with insufficient attention paid to whether service content matches users’ actual needs, whether access arrangements accommodate diverse groups, and whether participation processes are conducive to social interaction and emotional regulation. Under this logic, internal migrants are mostly regarded as service recipients rather than subjects of cultural participation, constraining the release of the mental health support potential that public cultural services inherently possess. Accordingly, the cultural participation opportunities, expression rights, and identity recognition needs of internal migrants should be incorporated into the framework of legal protection, and public cultural services should be transformed from general cultural supply into institutionalized public resources oriented toward mental health prevention.

## Introduction

In 2020, China’s internal migrant population reached 376 million, an increase of 154 million or 69.73% from 2010, accounting for approximately one-quarter of the total national population (1). The term “internal migrant” in this paper follows the operational definition from the Seventh National Population Census: individuals whose place of residence differs from the township or subdistrict of their household registration and who have been away from the registration location for more than 6 months, excluding those separated within the same municipal district. According to the China Population Census Yearbook 2020, among inter-provincial migrants, over 46 million have resided in the destination area for more than 5 years, with those residing over 10 years exceeding 20% (2). These figures indicate that internal migration has largely transitioned from short-term and transitional spatial movement toward a pattern of “stable mobility,” characterized by long-term residence, continuous employment, and repeated migration in cities (3). As internal migrants deepen their ties with host cities, their aspiration for settlement security intensifies. Yet persistent institutional disparities and inadequate social support prevent them from achieving this desired stability. The primary challenge has consequently shifted from short-term adaptation to chronic psychological distress, elevating mental health risks for this population (4, 5). While depressive disorder prevalence in the general Chinese population remains relatively limited, detection rates of depression, anxiety, and related psychological distress are consistently higher among internal migrants (6, 7). Migration experience, long working hours, occupational instability, income pressure, insufficient social support, and difficulties in integration are all significantly associated with elevated psychological risk. The long-term accumulation of psychological distress not only impairs individual quality of life, but also increases the burden of physical illness and the risk of mortality (8, 9).

However, access to formal mental health services is constrained by multiple factors, including the uneven resource distribution (10), high help-seeking costs (11, 12), compressed working hours (13) and the stigma of mental health problems (14). As a result, many internal migrants, even under considerable psychological pressure, cannot access the professional service system in a timely manner (15). This implies that the mental health challenges of internal migrants cannot be fully addressed by merely expanding formal medical supply; rather, more preventive, low-threshold, and low-stigma support mechanisms need to be sought at the level of everyday life (16–18). World Health Organization (WHO) review on arts and health points out that artistic and cultural practices play a positive role in health promotion, disease prevention and the management of acute and chronic diseases, and recommends that countries incorporate them into a broader health promotion framework through cross-sectoral cooperation (19). Relevant studies also show that there is a relatively stable correlation between cultural participation and multiple mental health indicators such as depression, anxiety, loneliness, cognitive decline, psychological resilience and social relationships (20, 21). For internal migrants, this low-threshold, low-stigma participatory practice embedded in daily life may precisely constitute a front-end buffer against psychological risks. For this reason, cultural participation should not be arranged only as a to-do item in the administrative supply system, but should be elevated to the content of claimable and guaranteeable rights and included in the vision of legal protection.

At present, relevant studies are generally carried out along two paths: One strand centers on equalization of public cultural services, which refers to promoting equal opportunities and roughly comparable quality of basic public services across regions, between urban and rural areas, and between migrant and local residents (22–24); the other discusses the accessibility, help-seeking behavior and structural barriers of mental health services for internal migrants from a public health perspective (25, 26). Although both strands have generated valuable findings, few studies have brought these two concerns together to examine whether cultural participation can serve as a routine and preventive resource for the psychological support of internal migrants within China’s public cultural service system. This paper addresses this gap by examining the normative foundations, practical obstacles, and institutional pathways through which public cultural services can support the mental health of internal migrants. It argues that the institutional focus of public cultural services needs to shift from facility coverage and project delivery toward the guarantee of participation opportunities, the confirmation of subject status, and the generation of supportive relationships. The normative foundation of this shift lies in a rights-based approach to cultural services.

### The applicable boundaries and limitations of the medicalized mental health service path

Current institutional arrangements for protecting the mental health of internal migrants in China remain dominated by clinical intervention, relying primarily on professional medical services and psychological counseling. While this approach is irreplaceable in diagnosing and treating manifested psychological disorders and mental illnesses, it is insufficient on its own. The WHO’s report on Social Determinants of Mental Health establishes that social, economic, and environmental factors critically shape mental health outcomes, driving the steady accumulation of psychological risk long before distress reaches clinical thresholds (27). These same structural factors simultaneously constitute direct barriers to accessing professional services (28, 29). Existing research consistently shows that internal migrants face a dual predicament of elevated mental health risk and persistently low help-seeking intentions, attributable to the compounding interplay of institutional constraints, economic costs, and cultural beliefs (30, 31).

At the institutional level, access to formal mental health services remains constrained by the household registration system, welfare distribution model, territorial governance structure, and related policy arrangements (32). Although reform has loosened the rigid binding between public services and registration status since 2014 (33), service acquisition still exhibits strong territorial characteristics. Residence permits carry limited rights, with significant regional variation in qualification recognition, service content, and guarantee levels (34, 35). Even long-term migrants may therefore fail to obtain stable public service support equivalent to that of local registered residents. Moreover, service availability does not depend solely on policy provisions. Studies show that internal migrants’ subjective eligibility assessments, expectations of procedural complexity, and unequal information access all influence help-seeking behavior (36). Thus, even formally lowered institutional thresholds may not translate into actual service utilization.

At the economic level, precarious employment and prohibitive help-seeking costs markedly diminish willingness to seek formal mental health services. Internal migrants, particularly migrant workers, commonly face long working hours, low income, weak occupational stability, and compressed daily routines (37, 38). The time, transportation, and financial costs of accessing psychological counseling directly conflict with work schedules, income constraints, and family responsibilities, forcing migrants to prioritize survival over professional care. Classic research on healthcare access has long established that utilization depends not only on service availability but also on affordability, accessibility, opening hours, and continuity of participation (39). For internal migrants, difficulty in accessing mental health services is shaped less by subjective unwillingness than by labor market status and opportunity cost structures.

Cultural and social psychological factors constitute a more hidden but equally important barrier. The identification, expression, and treatment of mental illness are never purely medical issues but are embedded in specific social and cultural contexts (40, 41). In China’s context, shaped by mental illness stigma and Confucian values of self-restraint and family reputation, people often express emotional distress through somatic symptoms (42, 43). Concerns about “face,” family honor, and widespread misconceptions about mental illness lead to the deliberate suppression or avoidance of depression and anxiety (14, 44). This cultural influence is particularly evident among internal migrants with fragile social support networks and marginal occupational situations. The result is the internalization of stigma and reinforcement of self-reliance, with internal migrants distinguishing between acknowledgeable emotions and hardships not easily disclosed, preferring to bear burdens alone rather than seek professional help.

**Figure 1:**
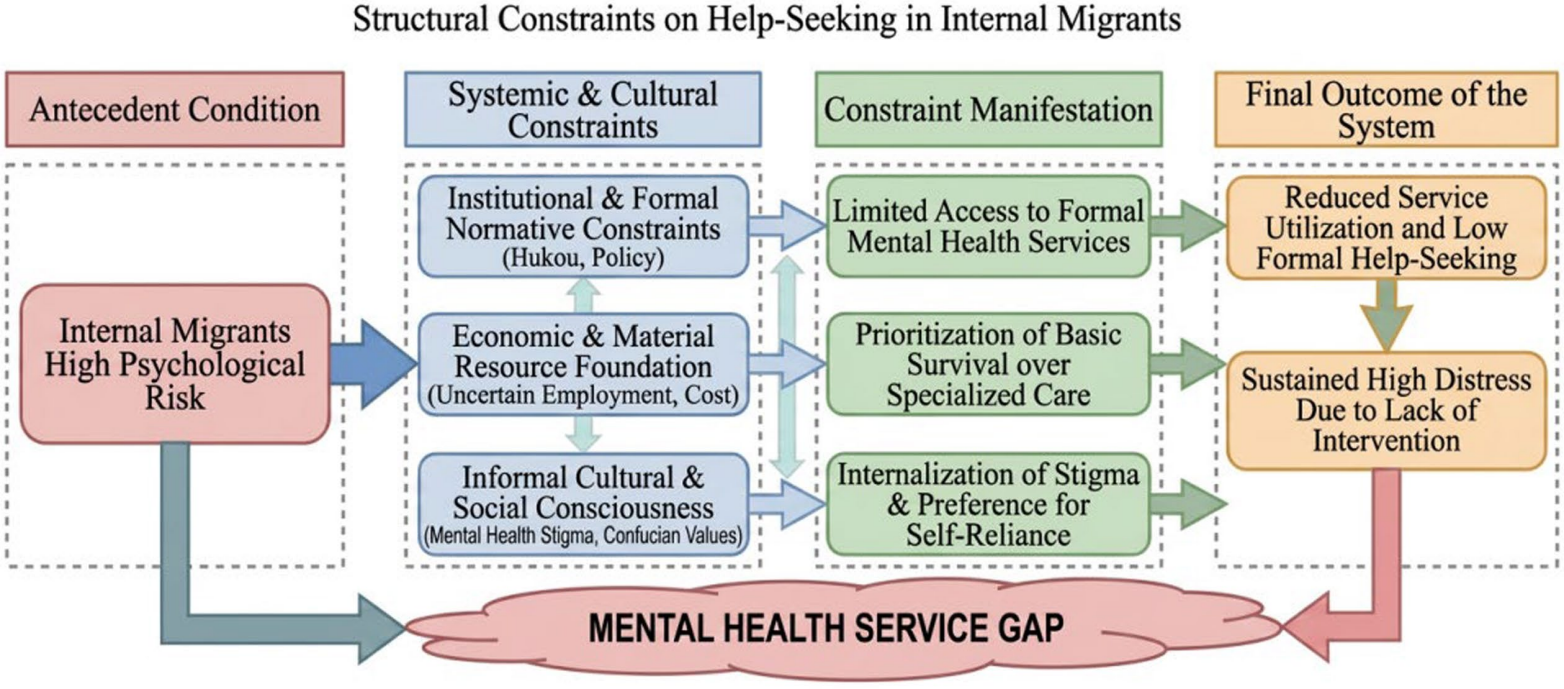
The Applicable Boundaries and Limitations of the Medicalized Mental Health Service Path. (Source: Authors’ own elaboration).

In summary, internal migrants, already bearing high psychological risk, are subjected to three mutually reinforcing systemic constraints: the institutional and formal normative constraints (Hukou and policy) limit their access to formal mental health services; the economic and material resource foundation (uncertain employment and high costs) forces a prioritization of basic survival over specialized care; and the informal cultural and social consciousness (stigma and Confucian values) fosters the internalization of stigma and a preference for self-reliance. These constraint manifestations intertwine, leading to reduced service utilization and low formal help-seeking at the individual level, and resulting in sustained high distress due to lack of intervention at the population level. This imbalance between high psychological risk and the underutilization of existing professional care ultimately culminates in a vast mental health service gap, which further exacerbates migrants’ initial high psychological risk through a self-reinforcing feedback loop (Figure 1).

Relying solely on the medicalized pathway centered on professional help-seeking and remedial intervention thus proves inadequate for addressing the large-scale, continuously accumulating, and predominantly non-clinical mental health risks faced by internal migrants. The potential of cultural participation as an upstream proposed pathway lies not in any claim that it displaces professional treatment, but in the recognition that its embeddedness in everyday life, accompanied by lower access thresholds and reduced stigmatization, enables individuals to access opportunities for emotional expression, social connection, a sense of belonging, and meaning-making outside clinical settings (45). Operating at the level of psychological resilience and risk buffering, this pathway intervenes before distress reaches clinical thresholds, functioning as a form of preventive support. This mechanism carries particular significance as an institutional complement for internal migrants. Accordingly, this paper will further examines whether institutionalized arrangements for public cultural services can provide internal migrants with a psychological support pathway that is at once accessible, continuous, and preventive in orientation.

### The normative basis for the health promotion function of public cultural services

Governments worldwide are increasingly incorporating arts and culture into health promotion and social cohesion policies to address loneliness, anxiety, and social alienation (46). China has also gradually attached importance to the supporting role of cultural participation in mental health, moving into a substantive promotion stage through National Center for Mental Health projects (47, 48), research initiatives (49), and blue book planning (50). However, policy advocacy and departmental guidance remain vulnerable to fiscal cycles, administrative priorities, and variable local implementation. These constraints make it difficult for public cultural services to sustain the normalized mental health protection that internal migrants require. It is therefore necessary to return to the existing legal framework and examine whether it provides sufficient normative grounding for public cultural services to genuinely assume this function.

The Public Cultural Service Guarantee Law of the People’s Republic of China, promulgated in 2016, constitutes the starting point for analyzing the health logic of public cultural services. As used in this article, “health logic” does not imply diagnostic or therapeutic functions, but is grounded in the efficacy of cultural participation in promoting mental health as established by empirical research (51, 52). On this basis, the state maintains the foundations of citizens’ spiritual life, conditions for social connection, and daily support networks through the institutionalized provision of cultural resources, thereby exerting promotive effects on psychological wellbeing (53). The function of law is to provide stable, predictable conditions for the association between culture and health, enabling public cultural services to serve as an institutional resource within the mental health prevention system rather than merely a general guarantee of cultural life. Although the Law does not expressly include “health” in its legislative purpose, this does not foreclose a normative connection between public cultural services and health promotion, its institutional structure reveals such a basis at three levels.

Legislative intent offers a clear anchor. Article 1 explicitly states that “this Law is enacted for the purposes of strengthening the development of the public cultural service system, enriching the spiritual and cultural life of the people.” “Spiritual and cultural life” refers to a state in which individuals maintain spiritual enrichment, obtain a sense of meaning, and develop the capacity for social participation. From a holistic health perspective, spirituality has been shown to play a significant role in promoting mental health and alleviating mental illness (54). Public cultural services are therefore required to sustain citizens’ spiritual vitality and sense of social belonging. For internal migrants, the impoverishment of spiritual life and the rupture of social ties following departure from familiar networks serve as a primary catalyst for mental health risk accumulation, compounded by financial precarity and job insecurity. By ensuring the accessibility of cultural resources, public cultural services may provide fundamental conditions for internal migrants to rebuild daily interactions and restore life order in unfamiliar cities.

At the mechanism level, Article 37 stipulates that “the State encourages citizens to actively participate in public cultural services and independently carry out healthy and civilized mass cultural and sports activities; local people’s governments at all levels shall provide necessary guidance, support and assistance.” The institutional language of “encourages,” “active participation,” and “guidance, support and assistance” establishes that public cultural services are a participatory public life practice rather than a one-way administrative supply. The normative implication is that the state is obligated not merely to provide cultural resources, but to facilitate citizens’ integration into public cultural life, enabling interactive communication and the formation of daily support relationships. Unlike volunteering, where social connections and belonging are generated through helping others (55), cultural participation achieves belonging and meaning-generation through the expression, interaction, and co-creation experienced in cultural practices. In collaborative creative activities such as theatre performance, choral singing, or craft-making, participants’ unique contributions—their voice, story, and embodied performance—are acknowledged as irreplaceable, and their subjectivity is validated through this process (56). For internal migrants, it is only when they are guaranteed the opportunity to participate actively as subjects, to build social connections through such participation, and to accumulate experiences of belonging that public culture can become a supportive resource for mental health rather than mere leisure consumption.

At the level of implementation, the equalization, convenience and universal benefit of public cultural services (57) give them important institutional advantages different from professional psychological services. Compared with the formal medical system, public cultural services are usually carried out relying on communities, blocks and other daily life spaces, characterized by low entry thresholds, reduced stigma, and flexible participation. These features are of particular practical significance for internal migrants who face difficulties in consistently accessing the professional mental health service system, as they provide a public support pathway that bridges the gap between formal medical treatment and private self-endurance.

The preceding analysis indicates that the legal framework already contains institutional resources for public cultural services to support mental health. Whether these institutional possibilities translate into tangible benefits for internal migrants, however, remains to be examined.

### The institutional gap and exclusion of internal migrants’ agency in public cultural services’ mental health causes

Normative interpretability does not automatically translate into practical realizability. Whether public cultural services can serve as mental health resources for internal migrants depends not only on statutory provisions, but also on whether these services in practice form a support network that is accessible, participatory, sustainable, and emotionally responsive.

**Figure 2:**
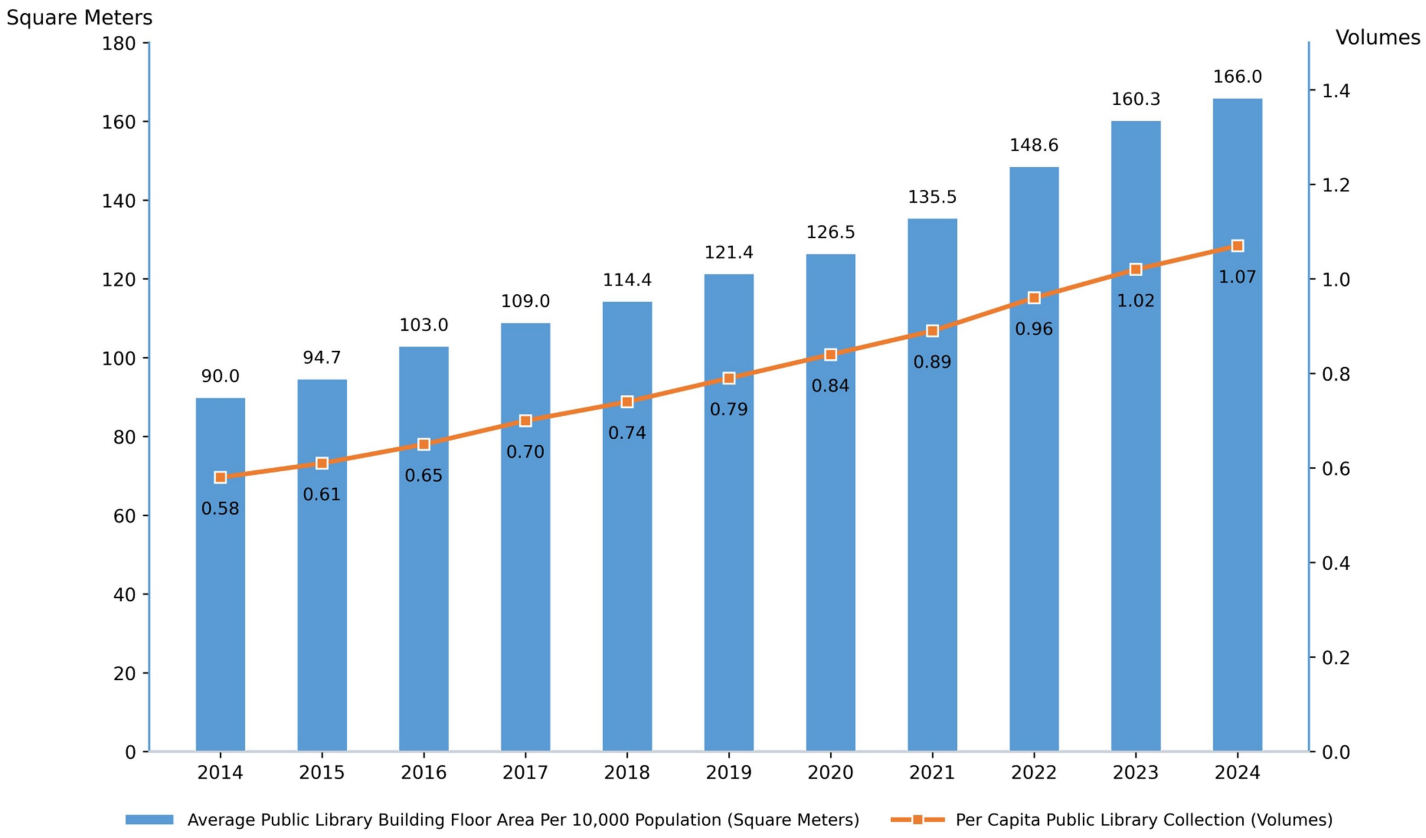
Per capita resources of public libraries in China, 2014–2024 (Source: Official Website of the Ministry of Culture and Tourism of the People’s Republic of China (58)). Adapted (translated from the original Chinese version into English; underlying data unchanged); Copyright © Ministry of Culture and Tourism of the People’s Republic of China.

**Figure 3:**
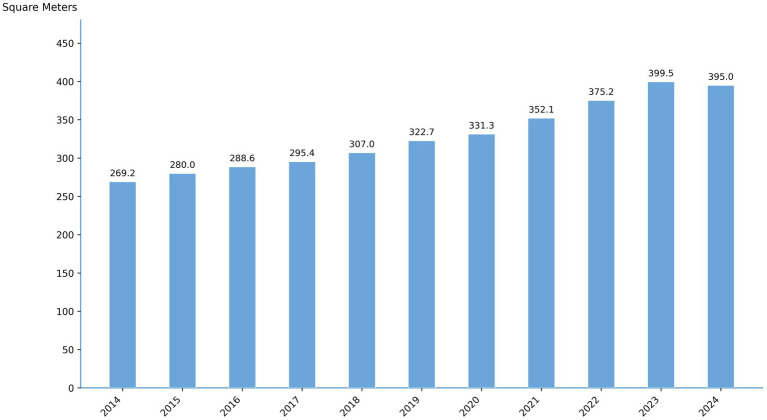
National average building area of mass cultural facilities per 10,000 people, 2014–2024 (Source: Official Website of the Ministry of Culture and Tourism of the People’s Republic of China (58)). Adapted (translated from the original Chinese version into English; underlying data unchanged); Copyright © Ministry of Culture and Tourism of the People’s Republic of China.

In practice, public cultural services have long been facility-oriented, and expansion of facilities has not necessarily improved service efficiency. The state has steadily increased investment in cultural infrastructure in recent years. By the end of 2024, there were 3,248 public libraries nationwide, with total floor space of 23.372 million square meters and per 10,000 people space of 166 square meters (Figure 2). There were 44,000 mass cultural institutions, with total floor space of 55.623 million square meters and per 10,000 people space of 395.0 square meters (Figure 3) (58). The Department of Public Services of the Ministry of Culture and Tourism also reported that the number of new public cultural spaces nationwide has exceeded 40,000 (59). Unlike traditional facilities, these new spaces are embedded in everyday living environments across urban and rural areas rather than concentrated in administrative centers, a pattern that may enhance physical accessibility for internal migrants. Yet physical accessibility does not guarantee service effectiveness. A more fundamental problem is that public cultural services have prioritized facility construction and performance evaluation, while the capacity of service content to deliver psychological support is far more critical for this population.

To be sure, facility expansion held foundational significance in the initial stage of institutionalization; without spatial carriers, a public cultural network cannot be established. However, scale expansion does not necessarily translate into improved efficiency (60). The law enforcement inspection reports from the Standing Committees of the National People’s Congress and local people’s congresses have repeatedly identified the persistent problem of “emphasizing facility construction while neglecting management and utilization” (61–63). This reflects a system dominated by “facility orientation,” where construction and evaluation focus on quantifiable indicators such as the number of venues, building area, equipment configuration, number of activities and service person-times, while overlooking real accessibility, usage sustainability, participation experience, emotional connection, and spiritual gain for service users (64, 65).

Accompanying the material orientation is a prominent supply-led tendency. Public cultural services have long followed a top-down administrative supply model, where grassroots cultural institutions organize services in response to government deployment rather than from service objects’ actual cultural life (66). The result is a structural bias: delivery-type, exhibition-type, and festival-type activities proliferate, while cultivation-type, companion-type, and community-type cultural construction remain underdeveloped (67); Standardized, easily quantifiable projects are favored in assessment systems, whereas practices requiring long-term investment but more responsive to individual spiritual needs and community connections receive insufficient support (68). The deeper issue is not merely supply methods but an implicit presupposition that predetermines what culture merits dissemination and which activities should be organized, rather than recognizing service objects as subjects capable of self-expression, self-organization, and co-shaping cultural life (69). Consequently, although public cultural services formally extend into grassroots areas, they may remain distant from the actual life rhythms of grassroots society. Such distance fundamentally constrains the activation of their psychological support function.

Some studies have employed Berry’s acculturation framework to analyze internal migrants’ social integration in China, examining their orientations toward “integration, assimilation, separation, and marginalization” (70, 71). This framework illuminates how internal migrants adjust between their original cultural identity and host city norms, offering a conceptual tool for identifying different integration paths (72). Yet its theoretical focus on how migrants adapt to existing social conditions rather than how they might transform their marginal position through cultural expression and institutional participation. Direct application of this framework would confine internal migrants to selecting from existing services rather than enabling them to create, organize, or negotiate cultural content based on their own experiences and needs. But meaningful psychological support requires more than cultural participation; it also depends on whether participants can gain experiences of self-expression, emotional connection, identity formation, and social recognition through that participation.

These gaps in institutional practice call for a fundamental reorientation of public cultural services toward spatial accessibility, subject participation, and daily relationship generation. Such a reorientation, however, is not merely a technical matter. It requires rethinking the organizing logic of public cultural services around rights.

### Reshaping the health logic of public cultural services with a “rights-based” approach

The “rights-based” approach constitutes a viable analytical framework for unlocking the health-promoting potential of public cultural services. It does not merely add a layer of rights discourse to the existing “facility provision” framework. It seeks to transform the fundamental logic through which public cultural services are organized and evaluated. This shift represents a paradigm change. Cultural services are no longer viewed as charitable gifts or welfare provisions granted by the state. They are recognized as enforceable legal rights—substantive rights to cultural participation. It should be noted that while this paper draws on studies of artistic activities as the primary supporting material, it does not reduce culture to art. Culture is a broader superordinate concept, and art constitutes only one of its components. The extensive use of artistic participation as the operationalization of cultural participation in evidence-based research does not imply equivalence between the two concepts. Artistic activities such as music, dance, and crafts inherently exhibit stronger characteristics of subjective engagement, creative expression, and emotional externalization. These characteristics form the core distinction between active cultural participation and passive cultural reception. For this reason, the empowering effect of artistic participation on mental health is more pronounced, and related research has accumulated most densely in this field (73, 74). This paper uses this body of evidence to analyze why active participation constitutes the critical condition for health effects, rather than narrowing cultural participation to artistic activities themselves.

**Figure 4:**
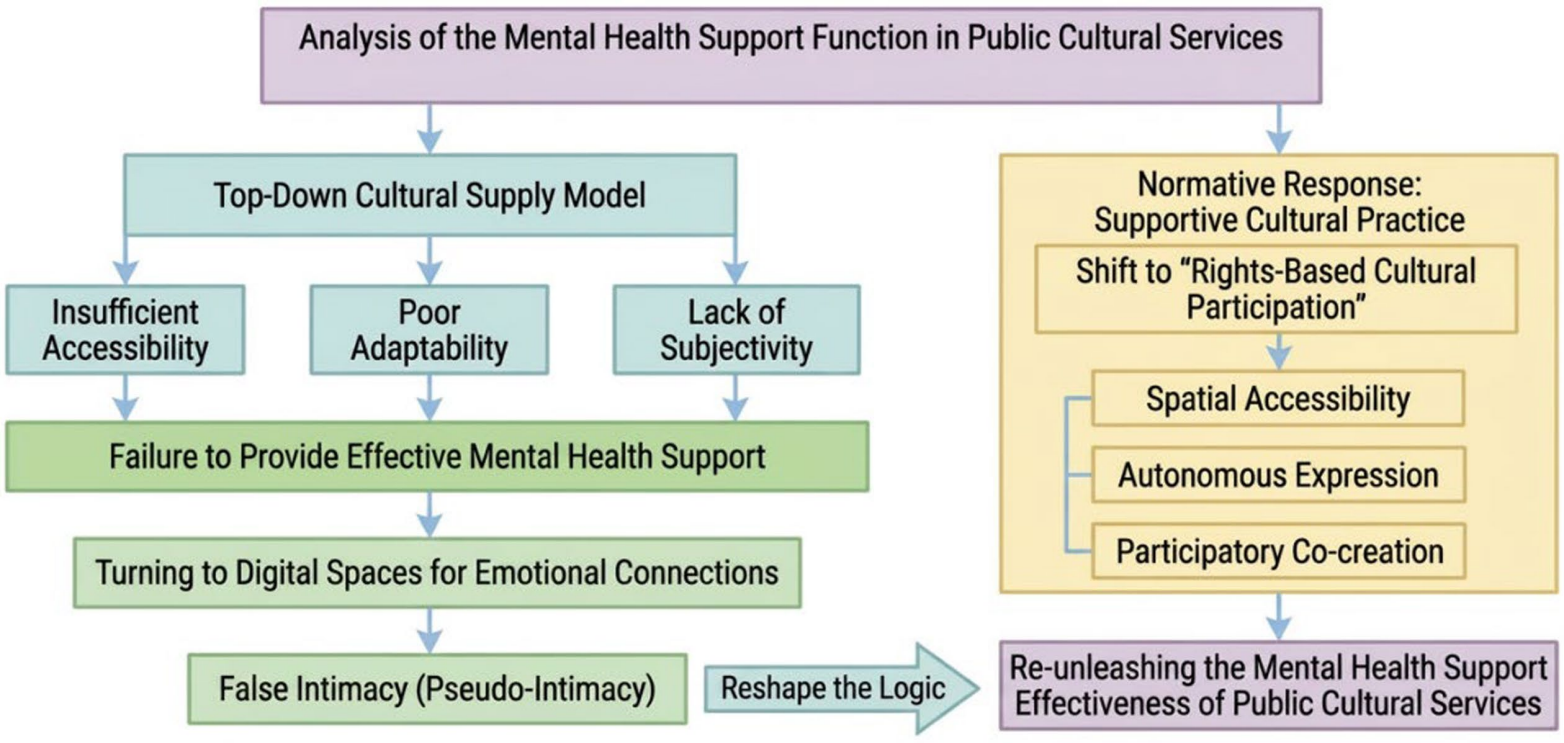
Reshaping the Health Logic of Public Cultural Services with a “Rights-Based” Approach. (Source: Authors’ own elaboration).

The traditional top-down model of cultural provision centers on venue construction, event delivery, and coverage rates. It overlooks citizens’ access possibilities, expressive needs, and relational construction as subjects of cultural practice. A rights-based perspective demands that the evaluation axis of public cultural services shifts from “whether facilities exist” to “whether participation is possible.” Realizing “participation possibility” requires meeting three sequential conditions: spatial accessibility, autonomous expression, and co-creative engagement (Figure 4).

Spatial accessibility is the first precondition for participation. The mere existence of physical infrastructure does not guarantee access to real opportunities for engagement. Research at the macro level documents significant geographic disparities in cultural participation. Simply increasing the number of facilities or events cannot automatically close the accessibility gap. Policies need to be calibrated to the distinct conditions of different groups (75, 76). The core practice elements identified by Goldstein and colleagues demand that cultural practices genuinely respect the life rhythms and cultural habits of diverse groups in decisions about facility location, opening hours, and content design (77). Translating these principles into institutional arrangements calls for dedicated implementation mechanisms at two levels. First, the needs and views of internal migrants should be incorporated into decision-making on the siting of public cultural spaces through community cultural councils or needs assessment mechanisms, with the extent to which their input has been taken up made part of the public documentation of the process, so that migrants hold a substantive right to participate in decisions about where facilities are placed and what functions they serve (78). Second, local regulations on the management of public cultural facilities should make evening and weekend staggered opening a binding requirement, so that the hours match the leisure time actually available to internal migrants (79). Compliance with this requirement should be built into the performance assessment indicators for public cultural services, turning what would otherwise be a hortatory provision into an accountable institutional demand.

Spatial accessibility addresses the question of “whether one can enter.” Whether individuals can express themselves as subjects once inside directly determines whether the health effects of cultural participation can be activated. A large body of empirical research has repeatedly confirmed that creative activities that call for active engagement and expressive creation, such as music, dance, and crafts, show more consistent associations with lower levels of anxiety and depression and higher life satisfaction. By contrast, receptive activities like viewing and listening, while not without some positive effect, mostly deliver short-term mood regulation rather than sustained psychological support (80–82). At the mechanistic level, the mental health benefits of culture depend on the involvement of subjectivity and agency, and it is precisely these deeper processes that receptive activities struggle to reach (83, 84). Reversing the supply-driven institutional inertia therefore has to start from the protection of procedural rights. On the one hand, the performance assessment system for public cultural services should be reformed so that indicators that reflect the participation of subjects, such as the rate at which needs assessments among internal migrants are conducted and the rate of their participation, are written into the statutory assessment criteria and carry a weight balanced against that of infrastructure-oriented indicators (85). On the other hand, a regular funding mechanism for cultural activities self-organized by internal migrants should be established, with simplified application procedures and lower eligibility thresholds, so that small-scale cultural activities initiated by migrants themselves receive stable, institutionalized resource support (86). This would turn autonomous expression from an occasional individual act into a right backed by institutional guarantees.

Autonomous expression secures the depth of individual participation in cultural practice, while co-creative mechanisms translate that individual expression into sustained social ties. For the internal migrant population, the fraying of social ties is itself a major source of mental health risk. As meaningful in-person interaction dwindles, migrants are pushed to seek substitute connections in digital spaces (87), yet such fragmented online interactions rarely provide genuine emotional support (88, 89). A number of studies confirm that the core mechanism through which participatory cultural activities contribute to mental health lies in the bonds participants build with one another during collaborative creation (90, 91), bonds that are activated through processual elements such as active engagement, self-expression, and peer interaction (92), and that deepen through emotional resonance, friction, and exchange in a shared activity space (93). The integrative review by Sonke and colleagues further demonstrates that physical co-presence in community spaces, culturally rooted program design, and the building of social relationship networks are the key social mechanisms that translate cultural participation into mental health outcomes (94). This finding carries direct implications for institutional design. The reason top-down, standardized cultural provision struggles to turn into mental health support for internal migrants is precisely that it bypasses the crucial link of co-creation (95). Institutionalizing co-creative mechanisms requires shifting the role of government from a direct provider of cultural content to a builder of the field of cultural interaction. At the operational level, public cultural institutions should be encouraged to cooperate with community organizations and social work agencies, nurturing the capacity for cultural self-organization among internal migrants by offering resource support, such as venues, equipment, and basic guidance, while refraining from interfering with the content and organizational forms of their activities (96). Only in this way can public cultural services move from one-way delivery to a public space that internal migrants themselves co-write and in which they build mutual ties.

## Conclusion

The value of public cultural services to the mental health of internal migrants lies not in the existence of cultural resources themselves, but in whether internal migrants can enter public cultural life as subjects and form the experience of expression, connection, belonging and recognition in participation. Whether public cultural services can support mental health is therefore better understood as an institutional question contingent on the protection of participation rights, rather than a direct consequence of cultural supply in itself. The combined effects of facility orientation, supply dominance, and insufficient subjectivity in the current practice makes it difficult for public cultural services to be transformed from general cultural supply into a stable mental health support mechanism. Correspondingly, the key to reconstructing public cultural services is not simply to increase venues and activities, but to carry out institutional adjustments around participation opportunities, relationship security and subject status.

### Limitations of the study

This study has certain limitations that should be addressed in future research. First, this paper does not use primary data to empirically test the causal mechanism between public cultural participation and mental health, so the discussion on "health effects" is mainly based on normative analysis and the synthesis of existing studies. Second, this paper has not subdivided the differentiated needs of different subgroups of internal migrants, so the proposed institutional suggestions are mainly based on overall optimization. Third, this paper focuses on the Chinese legal context, and the treatment of cross-national institutional comparisons and local practice differences is still relatively limited. Follow-up research should continue to advance at the three levels of micro-participation mechanisms, group heterogeneity and comparative institutional analysis.

## Data Availability

The original contributions presented in the study are included in the article/supplementary material, further inquiries can be directed to the corresponding author.
